# Impact of internal brand management on sustainable competitive advantage: An explanatory study based on the mediating roles of brand commitment and brand citizenship behavior

**DOI:** 10.1371/journal.pone.0264379

**Published:** 2022-03-11

**Authors:** Fatima Nawaz Qureshi, Shahid Bashir, Asif Mahmood, Sheraz Ahmad, Saman Attiq, Muhammad Zeeshan

**Affiliations:** 1 Faculty of Management and Sciences, Shaheed Zulfikar Ali Bhutto Institute of Science and Technology, Islamabad, Pakistan; 2 Department of Business Studies, Namal University, Mianwali, Pakistan; 3 KMITL Business School, King Mongkut’s Institute of Technology Ladkrabang, Bangkok, Thailand; 4 Air University School of Management, Air University, Islamabad, Pakistan; 5 UCP Business School—University of Central Punjab, Lahore, Pakistan; University of Salento, ITALY

## Abstract

The existing literature on internal branding has often adopted a managerial-based approach and seldom considered employees’ perceptions. Therefore, there is a need to understand the perspective of frontline and non-managerial employees. In this context, the current study investigates the impact of internal brand management on brand commitment, brand citizenship behavior, and sustainable competitive advantage for the hotel industry. A survey-based quantitative data was gathered from 390 non-managerial frontline staff working in 3-, 4-, and 5-star hotels of Pakistan. The results revealed that internal brand management positively impacts brand commitment, brand citizenship behavior, and sustainable competitive advantage. Besides, brand commitment has a positive impact on brand citizenship behavior and sustainable competitive advantage. Moreover, brand citizenship behavior has a positive impact on sustainable competitive advantage. In addition, the mediating roles of brand commitment and brand citizenship behavior exist between internal brand management and sustainable competitive advantage. The research implications, together with research limitations, have also been discussed.

## 1. Introduction

Branding has proved to be the best trend abounding the hotel industry worldwide [1–3]. In contrast to product brands, due to discomfort, segregation, and diversity of services [4], customers’ perception of a hotel service relies heavily on workers’ performance [5]. Consequently, the employees’ efficient service delivery is essential for the success of hotel businesses, and, therefore, the concept of internal branding has been introduced explicitly for branding among employees [6]. Employees are the prime focus in internal branding because they are essential to build a brand and shape it inside the customers’ minds. Both scholars and practitioners agree that successful service-brand enactments are necessary for the acquisition of competitive advantage. Thus, businesses need to hire proficient workers in competitive spirits to enhance their performance. Accordingly, companies now focus their customers on aligning the external and internal brand management [7, 8]. Managers patronize their employees in different ways [9, 10], for example, by giving them a sense of belonging and building an atmosphere that encourages them to continue and participate [10].

Thus, hotels hire human and physical resources to meet their targets [9], and keep themselves competitive for their survival. According to the resource-based and competence-based view, intense competition stems from high resources or skills. The expertise is at the organizational level but is inextricably linked to people [11], meaning that employees are critical to strategies for enhancing success [12]. Thus, there is a need to look at strategies and use tools to deal with them [2]. Hiring skilled people and trained managers and building efficient and affordable resources are critical factors for successful hotels [9]. Therefore, internal brand management is an effective tool for building strong brands, which leads to achieving sustainable competitive advantage in terms of high market share, customer loyalty, and price premium. The sustainable competitive advantage gained through this way is usually hard to imitate for the competitors [13].

Brand Commitment and Brand citizenship behavior are often the results of internal brand management, and both provide the sources of sustainable competitive advantage. Brand commitment is a psychological and emotional attachment of an employee to the brand he/she is working for. It is a crucial driver for the success of many industries, especially the hotel industry [14]. In previous literature, brand commitment is proven as an antecedent of brand citizenship behavior. Employees’ brand citizenship behavior contributes to strengthening the brand and making it inimitable for its competitors [15]. [16, 17] studied internal branding and recommended conducting more studies for the hotel industry, for example, through studying the effect of internal branding on brand commitment and brand-related behavior. Many of the studies undertaken on internal branding are based on a managerial approach only, and researchers on this topic have often adopted a managerial-based policy and did not consider workers’ perception of internal branding activities [18]. Therefore, there is a need to focus on frontline employees and non-managerial employees [17].

Furthermore, the previous empirical studies [19, 20] only emphasized a limited number of constructs. Those studies have discussed internal branding outcomes on employees’ responses, such as loyalty and brand performance. Only a few researchers have focused on critical constructs such as brand commitment, brand citizenship behavior [21]. Consequently, investigation of antecedent(s) and the consequence(s) of internal branding is still at the initial stages; therefore, more scholarly contributions are needed in this domain [22–24].

Therefore, this study aims to utilize the concept of internal branding to create a link between marketing and human resources. In this context, the effect of internal brand management on brand commitment, brand citizenship behavior, and sustainable competitive advantage has been examined for the hotel industry. The expected outcomes of this will be significant, as the recent researchers [17, 25–27] suggests the empirically testing of the internal brand management and its impact on the different types of organizational outcomes. For instance [25], said that we need more research to integrate the various resources to provide a clear definition of the company’s sustainable competitive advantage. [26] suggest that the focus of researchers should be to explore how human capital resources lead firms towards attaining a sustainable competitive advantage. [27] argue that the outside-in perspective is necessary to complement the inside-out standpoint in line with the competence-based view.

The current study results would carry the importance of internal branding in the hotel industry and reflect the process through which it leads toward achieving sustainable competitive advantage. Moreover, it will highlight the role of brand commitment and brand citizenship behavior in achieving sustainable competitive advantage. Furthermore, it emphasizes that in this era of intense competition, the hotel industry needs to attain sustainable competitive advantage through internal branding focused on their unique, rare, and inimitable employees.

The rest of the sections have been structured as follows. The relevant literature has been presented in the next section. After that, hypotheses have been formulated, followed by the portrayal of methods. After that, the data were analyzed, and the results were discussed. Eventually, both theoretic and applied implications have been presented.

## 2. Literature review

Strategic managers have always focused on achieving sustainable competitive advantage, especially in highly competitive markets [2]. Accordingly, searching for causes of competitive advantage has often been the concern of the researchers [2, 8, 28–33]. According to [31], a firm achieves a competitive advantage when its economic profit exceeds the typical market earnings (comprising the firms doing the same business). [33] argued that the competitive edge depends on how successfully and effectively the available resources have been used to meet the customers’ demands. Competitive advantage has various aspects such as price, quality, and innovation. However, employees are the primary source of sustainable competitive advantage in the service industry [2, 34] because every company has different kinds of people with various psychological values. According to [29, 32], there are four measures (such as value, rareness, inimitability, and sustainability) through which the resources of a firm can contribute to creating a sustainable competitive advantage. Besides, in service firms, employees have a significant role in achieving competitive advantage, incredibly sustainable differentiators [8, 34, 35]. [36] also linked the knowledge, attitude, value of the human resource, and competitive advantage.

Similarly, internal brand management can be defined as a strategy, or a systematically organized process, which leads the employees toward an acceptable brand behavior [37]. According to [38], internal brand management is based on communicating the brand to the employees effectively, convincing them about the relevance and worth, and successfully connecting all kinds of jobs to deliver brand essence. [39] argued that internal communication is the leading cause of internal branding, while [40] contended that training is the most significant and essential dimension of internal brand management. Likewise [19], argued that an extensive emotional and cognitive training network is integral for internal branding. Some other recent studies [10, 41] affirmed internal communication, training, and transformational leadership as essential aspects of internal branding.

Likewise, brand commitment is a "psychological attachment of employees to their organization, which motivates them to put an extra effort to achieve the organizational goals" [42]. Brand commitment can be taken as identical to organizational commitment [43] and is a crucial driver of success for many industries, especially the hotel industry [14]. [13] introduced three aspects of brand commitment: obedience, identification, and internalization. However, they were unable to prove that brand commitment is a multidimensional construct. [44] also believe that the concept of brand commitment is composed of only affective commitment. [51] also argued that brand commitment is a uni-dimensional construct. According to [14, 45], the employees must be committed to a true differentiation of the brand. When the employees have a better understanding of the brand values, there is a strong prospect of their psychological and intellectual engagement [14, 46]. Internal branding endeavors for a shared and clear understanding of brand values across the organization, which leads to brand commitment [15]. So, committed employees can better fulfil the brand promise because they are psychologically attached to it [16, 17, 46].

Brand citizenship behavior is a voluntary and unofficial behavior of the employees, strengthening the brand [47]. This concept has emerged from organizational citizenship behavior [48, 49]–an unofficial and voluntary behavior that supports job performance. [48] personalized the concept of organizational citizenship behavior to brand citizenship behavior. According to them, the behavior of employees is usually brand-oriented, which eventually strengthens the brand. [13] suggested that brand citizenship behavior is beyond the scope of organizational citizenship behavior since it has several additional aspects such as willingness to help, brand enthusiasm, and propensity for further development. [50] also investigated other aspects of brand citizenship behavior: helping behavior, sportsmanship, and civic virtue. [51] also studied that brand endorsement, brand development, and brand compliance are the additional aspects of brand citizenship behavior. [48] suggest that brand citizenship involves all those extra-role behaviors of employees aligned with the identity and promise of the brand. A brand can be advanced and enhanced through brand citizenship behavior. If all the employees (irrespective of their roles and departments) exhibit brand citizenship behavior, the delivery of brand promise can indeed be pledged. All the brand elements that strongly affect the customers’ brand experience are associated with the employees [52], not only with the salespersons or customer service providers but also with the entire employees who contribute brand [13].

### 2.1. Theoretical reflection

The resource-based theory supports the relationship between internal branding and sustainable competitive advantage, while social exchange theory explains the relationship between internal branding, brand commitment, and brand citizenship behavior. Both approaches are related to human resources and employees because the resource-based view portrays a firm’s competitiveness resulting from its resources and links these resources with people [11]. Social exchange theory describes that organizations need to motivate their employees and create a mutual relationship between employees and organizations to achieve their positive behavior and fulfill their organizational goals [53]. [54] proposed a relationship between internal branding, brand citizenship behavior, and customer satisfaction. They applied the social exchange theory as a base of their model. Similarly [51], explained the relationship between internal branding and its outcomes, such as brand commitment and brand citizenship behavior, with the help of a resource-based view. Thus, the current study has employed both.

### 2.2. Internal brand management and brand commitment

A total commitment is required to make a brand unique and thriving across all the employees of an organization [16, 45]. [49] suggest that internal branding results in brand commitment. Several other researchers [17] also suggest that brand commitment is a potential outcome of internal brand management.

Previous researchers [16, 55] observed that internal marketing communication towards employees is as important as external marketing communication towards customers. While external brand communication increases brand awareness among customers, internal brand communication increases awareness and motivates employees to understand the meanings of a brand [56]. Similarly, the training not only improves performance but also affects the attitude of employees positively. Because of training, employees perceive that they are also an essential and considerable part of the organization [15]. When the employees better understand their brand values, they tend to show more psychological commitments [46]. According to [15], communication and training are significant predictors of brand commitment [15].

Investing in leadership development, primarily transformational leadership, is worthwhile and essential to promote employees’ volitional and positive work behaviors, such as organizational citizenship behavior [57]. Transformational leadership has been proposed as an essential element of internal branding [41], as leaders have a strong influence on the social identifications of their followers. Moreover, transformational leadership effectively connects the employee’s self-concept to the mission [58]. Affective commitment has been confirmed as the most significant predictor of positive work behaviors. [59] explained how transformational leadership results in an affective commitment. They discussed that affective commitment is a psychological aspect. Transformational leaders inspire with emotional appeal; for instance, they induce a vision to work collectively to accomplish their interests. These leaders are also keen to understand and fulfill the needs of employees. All the efforts of transformational leaders create a positive perception of the employees about their organization–which eventually develops a high level of commitment [60]. Therefore, it is hypothesized that:

H_1_: Internal brand management positively influences brand commitment

### 2.3. Brand commitment and brand citizenship behavior

[61] noted a consistency in the significant positive relationship between brand commitment and brand citizenship behavior. They concluded that a positive job-related attitude influences organizational citizenship behavior. [62] also noted that organizational citizenship behavior is an outcome of employees’ satisfaction and commitment to the organization. [63] conducted a study on salespeople and found partial evidence of a relationship between employees’ satisfaction and organizational citizenship behavior.

So, there is a consensus in the primary literature of organizational behavior [64, 65] and internal brand management [48] that employees, who are emotionally and psychologically committed to their brand, are more likely to exhibit brand-building behavior. Several researchers [43, 66] believe that employees’ brand commitment is a strong predictor of their brand-related behaviors. For instance [43], found brand commitment a significant antecedent of brand commitment in the retailing sector. Moreover [80] corroborated the relationship between brand commitment and brand citizenship behavior in the airline sector. Furthermore [66], researched the banking sector and confirmed a strong relationship between these two constructs. Nevertheless, the relationship between brand commitment and brand citizenship behavior has not been investigated in the hotel industry. Therefore, the following hypothesis has been proposed:

H_2_: Brand commitment positively influences brand citizenship behavior

### 2.4. Brand citizenship behavior and sustainable competitive advantage

Both researchers and practitioners agree that effective brand development is needed to precede a competitive advantage in the hotel industry. In contrast to product brands, customer perception of the hotel service type is dependent on the service delivery performance [5] due to discomfort, segregation, and service inequality [4]. Customer-employee interaction influences customer’s perception of the highest quality; customer’s perception of brand quality and value considers the quality of the customer-brand relationship [67].

To facilitate brand performance, the role of internal branding depends on the perception that an employee’s representative resources (e.g., knowledge and skill) can be used to provide an ongoing competitive advantage for the organization [67]. Thus, brand citizenship is a potential consequence of internal brand management [68]. The success of a brand strategy depends mainly on the role of employees as product specialists [69]. Consequently, employee behavior has a significant impact on how external audiences perceive and feel about the product in the hotel industry, thereby building a competitive advantage [15]. So, it can be assumed that brand citizenship behavior can lead to a competitive advantage. Therefore, it is hypothesized that:

H_3_: Brand citizenship behavior positively influences sustainable competitive advantage

### 2.5. Internal brand management and brand citizenship behavior

[48] suggested that internal brand management has three different levels. The first level focuses on the personal identity of the brand by recruiting and promoting the employees. The second level is associated with internal communication and brand awareness to strengthen the brand among employees. The final level is based on brand leadership, referred to as the employees who live with the brand. Through all these three levels, brand communication created through internal brand management results in brand citizenship behavior. Accordingly, several researchers have observed brand citizenship behavior as a consequence of internal branding. For instance [13], researched internal branding and found brand citizenship behavior a result of internal brand management. Similarly [68, 70], also confirmed that brand citizenship behavior is a potential outcome of internal brand management. Based on the given theoretical and empirical support, it is hypothesized that:

H_4_: Internal brand management positively influences brand citizenship behavior

### 2.6. Internal brand management and sustainable competitive advantage

Compliance with internal branding is the key to achieve a sustainable competitive advantage; for example, a motivating leader positively influences the internal branding through seeking to solicit knowledge and learning by creating an environment conducive to working conditions, promoting psychological and personal attention, creating ingenuity, and encouraging problem-solving decisions [71]. A focus on internal brand management is required to connect the behaviors that act under the brand [72]. Internal brand management is considered a potential tool to gain a sustainable competitive advantage [73]. It is because internal brand management can make it difficult for the competitors to imitate or threaten the brand position. Although professional marketers play an essential role in creating and maintaining a brand, other employees also play a strong role in creating a competitive advantage through branding. Hence, every employee contributes to the organization at different levels, but their contribution to building a strong brand is undeniable [13]. Therefore, it is hypothesized that:

H_5_: Internal brand management positively influences sustainable competitive advantage

### 2.7. Brand commitment and sustainable competitive advantage

According to [1, 49], a necessary condition to strengthen a brand is establishing a brand commitment. It is a vital factor that leads to brand citizenship behavior, which leads to brand strength. Combined with sufficient disposable resources and employee expertise, brand commitment creates and maintains brand citizenship behavior, achieving a significant brand-strengthening effect. For instance [74], examined the substantial and positive relationship between brand commitment and sustainable competitive advantage.

Brand commitment reflects the equal capacity for the psychological identification of an employee and his or her involvement with the organization [75]. In addition, it reflects a strong belief in the organization’s goals: a willingness to make great efforts and a strong desire to retain [76]. Furthermore, it has a binding effect on learning-oriented and innovation-oriented competitive advantage relationships. Thus, brand commitment is likely to have a positive relationship with a sustainable competitive advantage. Based on the given theoretical and empirical support, it is hypothesized:

H_6_: Brand commitment positively influences brand sustainable competitive advantage

### 2.8. Mediating effects

When employees have understandable brand values, they engage with the brand psychologically and emotionally [46]. Brand commitment has been taken as a mediator in various studies and contexts. For instance [77], surveyed franchisor support and franchisee-perceived brand image and found that brand commitment partially mediates the relationship between franchisor support and franchise perceived brand image. [13] also proposed brand commitment as a vital mediator between internal branding and its outcomes. [43] also, while conducting a study to identify the determinants of brand citizenship behavior, concluded that internal branding is an antecedent of brand commitment, and brand commitment is an antecedent of brand citizenship behavior. They found that internal branding and brand commitment both are significant and strong antecedents of brand citizenship behavior. Another study [78], conducted in entrepreneurship, also showed an effective mediation of brand commitment between brand trust and brand citizenship behavior. Based on the given theoretical and empirical support, we propose:

H_7_: Brand commitment mediates between internal brand management and brand citizenship behavior.

Brand citizenship behavior is a potential consequence of internal brand management [68]. The successful branding strategy depends mainly on the role of staff as product specialists [69], especially in the hospitality industry. In this field, employee behavior significantly impacts how external audiences interact with a brand and, in addition, enhances competitive advantages [13]. So here, it can be stated that brand citizenship behavior can lead to competitive advantage. Brand citizenship behavior has been considered a significant mediator in the research frameworks of the studies conducted in the hotel industry [79].

Furthermore, it has been examined that brand citizenship behavior partially mediates the relationship between franchisor support and franchisee-perceived brand image [77]. The most admired model for internal branding presented by [13] considered brand citizenship behavior as a mediator between brand commitment and brand strength. Similarly [67], researched the hotel industry using a case study approach and applied a mixed-method (qualitative and quantitative) approach. They focused on frontline employees and their perception of internal branding. The authors concluded that brand commitment mediates between internal branding and brand performance. Based on the given theoretical and empirical support, it is proposed:

H_8_: Brand citizenship behavior mediates the relationship between brand commitment and sustainable competitive advantage.

[48] noted that internal brand management creates a brand commitment, leading to brand citizenship behavior. In addition [80], argued that brand commitment and brand citizenship behavior are required for internal brand management and examined that brand commitment contributes to brand citizenship behavior. In addition, employee morale has a significant impact on how external audiences interact with a brand and, by extension, build sustainable competitive advantages [13]; it can be assumed that brand citizenship behavior can lead to competitive advantage. Therefore, brand commitment and brand citizenship behavior depend on the perception that employees’ representative resources (e.g., knowledge and skill) can provide a sustainable competitive advantage to an organization [67].

Upon these mentioned propositions, the Hierarchy-of-Effects model can be utilized to understand the cascading effect. Brand commitment and brand citizenship behavior represent the employee’s internal process resulting from employees’ evaluation of the internal brand management, leading to the achievement of the firm’s sustainable competitive advantage. In other words, internal brand management of the hotels is unlikely to influence the sustainable competitive advantage unless employees’ clear brand commitment and brand citizenship behavior occur. Consequently, there is a likely case to consider brand commitment and brand citizenship behavior as a combined mediator between internal brand management and sustainable competitive advantage. Based on the given theoretical and empirical support, it is hypothesized:

H_9_: Brand commitment and brand citizenship behavior sequentially mediate the impact of internal brand management on sustainable competitive advantage.

**Fig 1:**
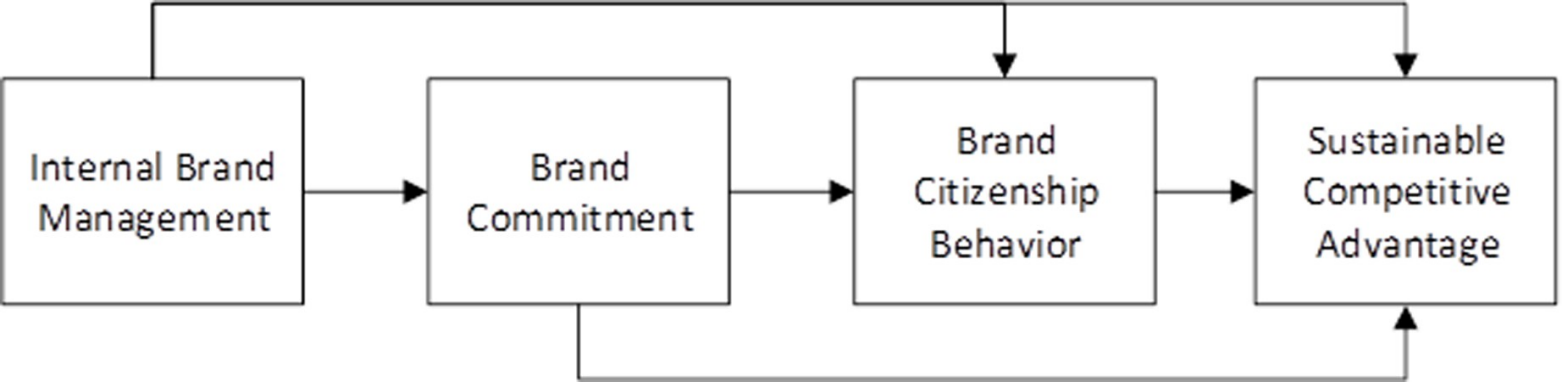
Theoretical model.

The hypothesized relationships have been depicted in Fig 1.

## 3. Methodology

### 3.1. Sample and procedure

Since the research objective was to examine the impact of internal brand management factors on sustainable competitive advantage, primary data from the participants were collected through a structured questionnaire using a quantitative approach. The respondents of this study were informed about the purpose of data collection. It was ensured that the collected data would be kept confidential and only be used for this research. The properly structured consent forms were distributed enclosed by envelope along with the questionnaire.

The population for this study was the non-managerial or frontline staff working in 3-, 4- and 5-star hotels of Pakistan. The frontline staff is usually in direct contact with customers, known as customer-interface employees, so it is essential to understand their perspective about internal branding and competitive advantage [34]. Data were collected from 14 hotels in Rawalpindi, Islamabad, and Lahore. Among these hotels, only two hotels were 5-stars, eight were 4-stars, and four were from 3-stars. Moreover, only four departments were selected to meet the purpose of the study: food and beverages, frontline office, housekeeping, and marketing departments [56].

Furthermore, purposive sampling was chosen to focus on people with particular characteristics compatible with the research purpose [81]. Four hundred and nine questionnaires were returned out of 700 distributed among the participants. After scrutinizing the received questionnaires, 20 were discarded because these were not filled correctly. Thus, 390 questionnaires were used for data analysis. Table 1 shows the demographics of the respondents. Out of 390 respondents, 268 (68.7%) were males, and 122 (31%) were female employees. Among the respondents, 10.8% of people had experience above five years, most of them had experienced between 1 to 3 years (41.3%), while 26.2% had experienced less than one year, and only 21.8% were in the category of 4 to 5 years of experience.

**Table 1:** Demographics of the respondents (N = 390).

		Frequency	Per cent	Cum. Per cent
Gender	Male	268	68.7	68.7
	Female	122	31.3	100.0
Experience	Below 1 year	102	26.2	26.2
	1–3 years	161	41.3	67.4
	3–5 years	85	21.8	89.2
	Above 5 years	42	10.8	100
Education	Intermediate	289	74.1	74.1
	Bachelor	91	23.3	97.4
	Masters and Above	10	2.6	100
Departments	Front office	123	31.5	31.5
	Housekeeping	133	34.1	65.6
	Food and beverages	96	24.6	90.3
	Marketing	38	9.7	100

Since the data were collected from non-managerial staff, the education level was comparatively less, as 74.1% of respondents were intermediate. According to Table 1, 23.3% of people had bachelor’s degrees, and only 2.6% were masters and above. Moreover, 34.1% of employees were from the housekeeping department, and 31.5% were from the front office department, 24.6% of employees participated from the food and beverages department, and only 9.7% of the marketing department participated in this survey.

### 3.2. Ethics statement

Ethical review and approval were obtained for this study on human participants from the committee named as ‘Ethical Research Committee’ of the Institution (NML-ERC/2020-014). Moreover, the participants provided their written informed consent to participate in this study.

### 3.3. Measures

According to [67], internal branding (IBM) comprises internal communication and training. [82] also considered only these two aspects. As [20] reported in their study, there is a dearth of proper and well-accepted conceptualization and operationalization of IBM, so various IBM dimensions have been considered in previous studies. A recent research study conducted on internal branding in the hotel industry by [41] operationalized IBM with three aspects: internal communication, training, and transformational leadership. The current study has also followed [41] to operationalize this construct, assessed by 13 items. The reported value of Cronbach’s alpha was 0.93.

Initially [48], introduced seven aspects of Brand Citizenship Behavior (BCB), but only three elements: willingness to help, the propensity of further development, and brand enthusiasm were finally confirmed as significant dimensions. Later on [79], also considered only these three aspects to form the construct of BCB. This research is also focused on only these three aspects of BCB. BCB was measured by 14 items adopted from [66]. The Cronbach’s alpha for these items was 0.93. Similarly, Brand Commitment (BC) has been operationalized as a one-dimensional construct, and only practical commitment has been considered in this study [80]. Likewise, brand commitment was measured by five items adapted from [66]. Cronbach’s alpha was 0.90.

A hotel’s competitive advantage is referred to as better services and performance than its competitors in the market [83]. In this study, sustainable competitive advantage has been operationalized as differentiated services, superior quality, unique benefits, and advanced services [84]. Four items were used to assess sustainable competitive advantage adopted from [83]. The Cronbach’s alpha for these items was 0.89.

## 4. Data analysis and results

### 4.1. Descriptive analysis

Table 2 exhibits descriptive statistics of the data. Skewness and Kurtosis values were evaluated to check the data normality. The skewness values of IBM (-.971), BC (-.747), BCB (-.309), and SCA (-1.534) are within the acceptable range. Similarly, the values of kurtosis for IBM (.522), BC (0.181), BCB (-1.037), and SCA (-1.828) are within the acceptable range, which confirms the normality of the data [85].

**Table 2:** Descriptive statistics.

Constructs	N	Mean	Std. Deviation	Skewness	Kurtosis
IBM	390	3.4900	.92724	-.971	.522
BC	390	3.7089	.92028	-.747	.181
BCB	390	3.3645	1.01361	-.309	-1.037
SCA	390	3.6117	.93393	-1.534	1.828

The mean value shows the center of data as many statistical analysts consider it a standard measure of central distribution of data. The mean value of IBM (3.89), BC (3.36), BCB (4.09), and SCA (-3.75) show the central distribution of data for these respective variables.

### 4.2. Measurement model

**Fig 2:**
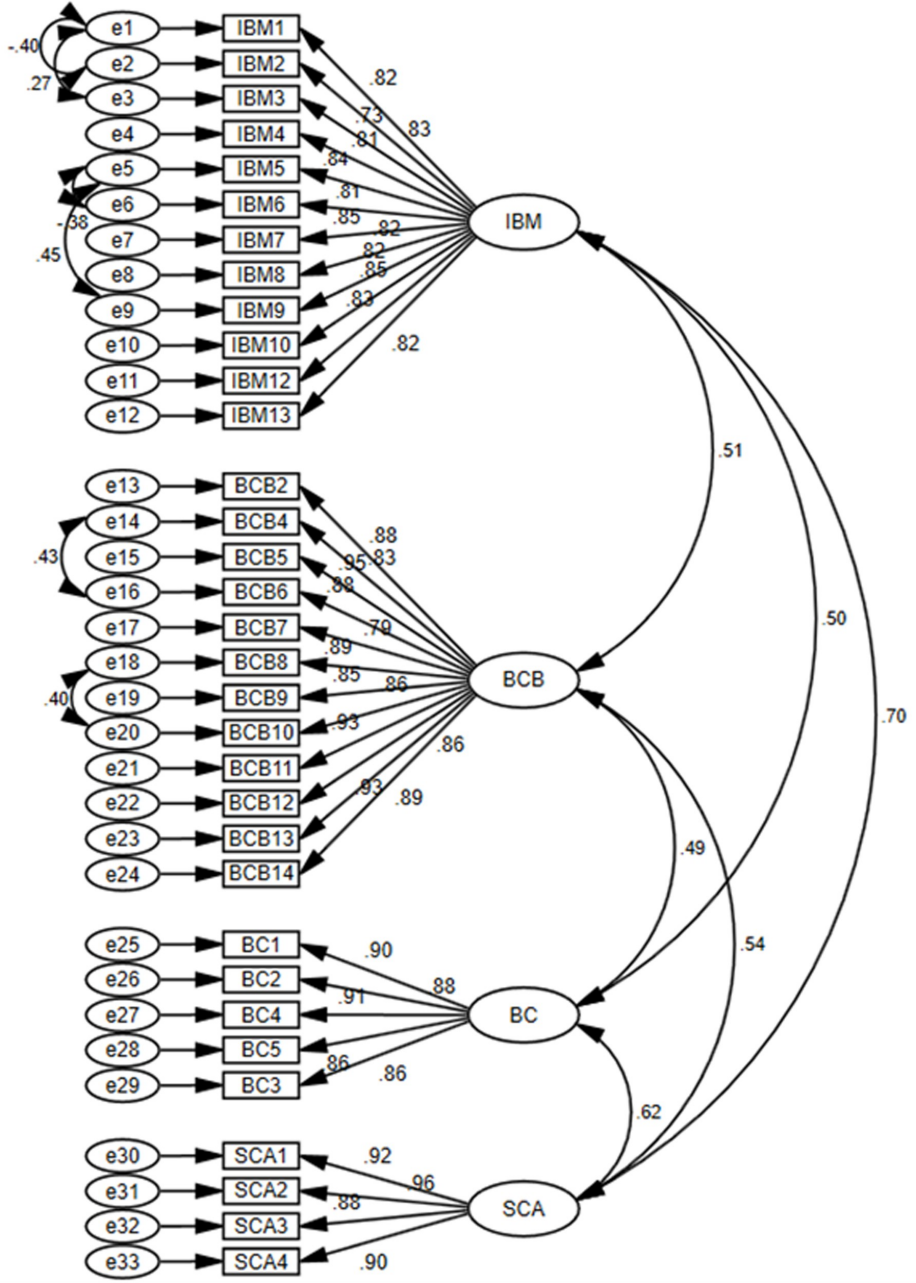
Confirmatory factor analysis (χ^2^/df = 2.737; IFI = 0.944; TLI = 0.931; CFI = 0.944; RMSEA = .067; SRMR = .0372).

The measurement model is used to assess errors in evaluating the theoretical concepts. Confirmatory Factor Analysis (CFA) was used to analyze the factor structure of the model. The estimation method maximum likelihood (ML) was used to check the loading of items on the corresponding factors, as shown in Fig 2.

Different model fit indices were used to check how data fit the theoretical model. The ratio of chi-square to its degree of freedom (CMIN/DF) was computed as an essential criterion of model fitness. The ratio of χ^2^/df = 2.737<3 indicates a well-fitted model. Similarly, the value of SRMR = .0372 is within the acceptable range (0 <SRMR < .08) [83, 86]. Whereas RMSEA value between 0.08 to 0.10 provides a mediocre fit and below 0.08 shows a good fit [87, 88]. The present research study results showed that the value of RMSEA = .067 is within the acceptable threshold. Furthermore, the results also showed that the value of NFI = .919, which is above the threshold of NFI >.90) [87]. In addition, the results also indicate that the value of CFI = .944 is within the acceptable range. The studies have shown that a value greater than 0.90 is required to avoid miss-specified models [89, 90].

The construct validity was established through convergent and discriminant validities. The convergent validity ensures whether the items load on the corresponding factors. On the other hand, discriminant validity measures how the items are different from other factors. The average variance extracted (AVE) values in Table 3 are above 0.5, proving that there is no issue of convergent validity. Similarly, discriminant validity was established because square roots of AVE values (shown in the diagonal) of all constructs were greater than the inter-construct relations [91, 92]. Moreover, the values of maximum shared variance (MSV) are less than the respective AVE values, and maximum reliability [MaxR(H)] is more significant than composite reliability (CR), which confirms that there are no concerns for discriminant validity. Additionally, Heterotrait-Monotrait Ratio (HTMT) analysis, shown in Table 4, confirms discriminant validity because the correlations between pairs are less than the threshold of 0.85 [91].

**Table 3:** Discriminant validity.

	CR	AVE	MSV	MaxR(H)	IBM	BCB	BC	SCA
IBM	0.961	0.674	0.492	0.962	0.821			
BCB	0.976	0.775	0.292	0.980	0.509***	0.881		
BC	0.947	0.781	0.384	0.948	0.497***	0.489***		
SCA	0.953	0.835	0.492	0.960	0.702***	0.541***	0.620***	0.914

***p<0.001, IBM = Interbal Brand Management, BCB = Brand Citizenship Behavior, BC = Brand Commitment, SCA = Sustainable Competitive Advantage.

**Table 4:** HTMT analysis.

	IBM	BCB	BC	SCA	Factor Loading Range	Cronbach’s alpha
IBM					0.735–0.852	0.950
BCB	0.506				0.795–0.952	0.975
BC	0.503	0.489			0.858–0.908	0.947
SCA	0.717	0.543	0.624		0.903–0.958	0.952

Furthermore, the value of Cronbach’s alpha reflects internal consistency (reliability). It measures how closely the items of a group are related to each other. [93] stated that alpha values must be more than or at least equal to 0.7. As can be seen from Table 4, the alpha values of all constructs are above the threshold value.

### 4.3. Structural model

**Fig 3:**
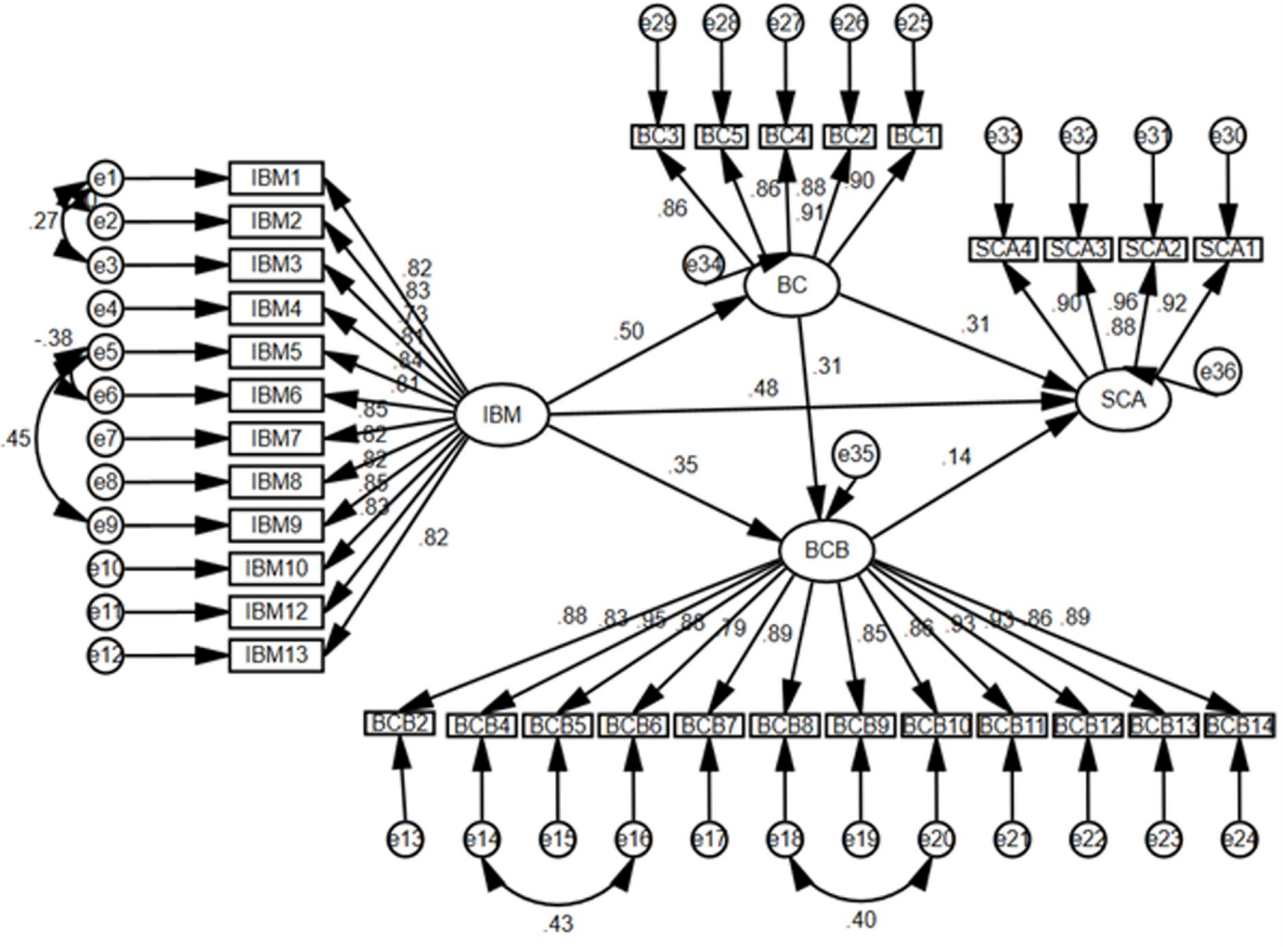
The structural model.

After confirming construct validity and reliability, the structural model was analyzed to test the hypotheses, as depicted in Fig 3.

H_1_, H_2_, and H_3_ are concerning the direct relationships of adjoining variables. The results (β = .532, p < .001; β = .286, p < .001; β = .154, p < .001, respectively) demonstrate that these hypotheses have been accepted related to variables of internal branding, brand commitment, brand citizenship behavior, and competitive advantage. Similarly, H_4_, H_5,_ and H_6_ are related to the direct relationships between variables bypassing the adjoining variables. The findings (β = .504, p < .001; β = .348, p < .001; β = .312, p < .001, respectively) confirm these hypotheses. The summary of details is presented in Table 5.

**Table 5:** Structural model evaluation.

Hypothesis	Path	Estimate	SE.	C.R.	p-value
H_1_	BC <—IBM	.532	.054	9.812	***
H_2_	BCB <—BC	.286	.048	5.929	***
H_3_	SCA <—BCB	.154	.046	3.334	***
H_4_	SCA <—IBM	.504	.050	10.021	***
H_5_	BCB <—IBM	.348	.052	6.681	***
H_6_	SCA <—BC	.312	.044	7.089	***
H_7_	BCB<—BC<—IBM	.156	[LCL = .110, UCL = .203]		***
H_8_	SCA<—BCB<—BC	.046	[LCL = .110, UCL = .203]		***
H_9_	SCA<—BCB<—BC<—IBM	.156	[LCL = .110, UCL = .203]		***

***p<0.001.

Likewise, hypotheses related to mediation effects, i.e., H_7_, H_8,_ and H_9,_ have been accepted (β = .156, p < .001; β = .046, p < .001; β = .156, p < .001, respectively), indicating the partial mediations. The significance of these relationships was computed by applying the bootstrapping procedure in AMOS [94].

## 5. Discussion

This research examines the impact of internal branding on brand commitment, brand citizenship behavior, and sustainable competitive advantage. It has been observed that internal branding is a strong predictor of brand commitment. The relationship between both variables is significant and positive (β = .532, p < .001). The results are consistent with previous studies such as [15, 70]. In this study, the critical factors of internal branding were communication and transformational leadership. In their meta-analysis [59], also concluded that transformational leadership leads to high employee psychological commitment.

Similarly, the results showed a positive relationship between brand commitment and brand citizenship behavior (β = .286, p < .001). This relationship has also been reported positive in previous research [43, 62]. The empirical findings of brand citizenship behavior and sustainable competitive advantage have not been discussed in the literature, but in previous research, conceptual relationships have been discussed to some extent. [13, 15] mentioned that employees’ behavior significantly influences developing competitive advantage in the hospitality industry. The current study provided empirical evidence that brand citizenship behavior is a solid and significant predictor (β = .154, p < .001) of sustainable competitive advantage, and the relationship between both variables are positive. Likewise, the results (β = .348, p < .001) indicate that the relationship between internal branding and brand citizenship behavior is significant; which is consistent with previous research [48, 68].

The relationship between internal branding and sustainable competitive advantage shows a strong, positive, and significant relationship (β = .312, p < .001). Though the previous research theoretically discussed that internal branding is a potential route to acquiring sustainable competitive advantage [95], the current study is the first study to provide empirical evidence. Similarly, the relationship between brand commitment and brand citizenship behavior is significant (β = .312, p < .001), agreeing with the previous investigations [43, 78].

Regarding the unique contributions of the current study, the results confirm brand commitment as a significant mediator between internal branding and brand citizenship behavior. Similarly, the mediating role of brand citizenship behavior between brand commitment and sustainable competitive advantage has been tested. The results showed brand citizenship behavior as a significant mediator between brand commitment and sustainable competitive advantage. In previous research, brand citizenship has been considered a mediator between brand commitment and brand performance and internal branding and customer satisfaction. In both studies, brand citizenship behavior has been proved as a positive and significant mediator [43, 79, 95]. Finally, concerning the sequential mediation results, the indirect impact with the mediation of brand commitment and brand citizenship behavior contributes to the beta value of .156 and p < .001. Since direct and indirect relationships are significant in all instances, partial mediations exist between the variables.

## 6. Implications

### 6.1. Theoretical implications

Organizations dealing with generic products or services do not focus on the internal branding of employees but on price competition. But since the hotel industry has the potential to provide differentiated services, it can get the advantage of sustainable competitiveness through internal branding to earn above-average profits. However, there is very little knowledge available on internal branding in the literature, and, furthermore, this concept requires empirical evidence to confirm the consequences of internal branding. In this regard, the current study contributes to the literature by providing empirical evidence for the relationships between the variables not studied before, such as internal brand management (IBM) and sustainable competitive advantage (SCA) in a hotel setting. Moreover, brand commitment (BC) and brand citizenship behavior (BCB) as sequential mediators, integrating them between IBM and SCA, have also been discussed and empirically proved, which were not tested in previous research. In other words, internal branding not only contributes to sustainable competitive advantage directly but also stimulates brand commitment and brand citizenship behavior of hotel employees, which in turn help establish SCA. Hence, IBM, BC and BCB are the fundamental constituents of SCA.

[25] recommend that there is a need to research firms’ competitive advantage using resource-based theory. The findings of this study confirmed the conceptualization of resource-based theory, implying that branding can be a source of competitive advantage. Based on this theory, this study proposed internal branding as a predictor of sustainable competitive advantage. However, researchers have overlooked this relationship, and there was no empirical study conducted to relate internal branding and sustainable competitive advantage. Following [25] recommendation, this study provides evidence that HRM practices lead to sustainable competitive advantage, as internal branding is part of HRM practices.

Furthermore, previous studies on managerial staff, capital, machinery, and monetary assets have been taken as a sustainable competitive advantage. In contrast, this study followed the different parameters [83] to measure sustainable competitiveness through the perception of non-managerial staff of hotels. Most interestingly, this study considered different dimensions of internal branding while testing the internal branding. As internal branding is a relatively new concept, researchers are still conducting exploratory analyses to determine its dimensions and antecedents. This study focused on three dimensions such as training, internal communication, and transformational leadership.

Similarly, in previous research, there was an argument that whether the brand commitment is uni-dimensional (affective commitment only) or multidimensional (additionally- normative and continuance commitment). By following the recommendations of [51], this study supports that brand commitment is a one-dimensional construct (affective commitment). As brand citizenship behavior is derived from organizational citizenship behavior, researchers are still unsure about its dimensions. Various researchers considered different dimensions, but the results were not consistent. In this study, three dimensions: willingness to help, brand enthusiasm, and the propensity of development [13], have been emerged as significant dimensions of brand citizenship behavior.

### 6.2. Managerial implications

The findings of the study provide managerial insights to hoteliers to create and sustain a competitive advantage. The sustainable competitive advantage of functions is heavily dependent on intangible strategic assets such as service employees. Internal brand management is particularly relevant in the hospitality industry due to the inconsistent nature of demand and service quality in the hospitality industry. A company’s competitive advantage helps gain more profits from the shareholders. Similarly, creating a sustainable competitive advantage is the most crucial goal of any organization and perhaps the most critical quality that requires the attention of any organization. This study showed how internal brand activities could help brands gain sustainable competitive advantage. The organizations that emphasize their employees’ internal branding stimulates brand commitment and brand citizenship behavior to generate sustainable competitive advantage ultimately.

Hotel managers should consider non-managerial frontline staff valuable internal stakeholders. Internal branding cannot be thought of in isolation, for example, at the time of recruitment, the employees who have fit with internal branding programs should be hired. Marketers and HR department need to work on closely the internal brand management system through daily briefing, group meetings, internal newsletters, notice boards, videos, blogs and training & development to achieve better consequences of IBM. Furthermore, close coordination among employees would create a sense of ownership to strengthen team spirit and internal branding. Moreover, managers should measure perceptions of brand and behavior of employees through continual in-house market research and take customized remedial actions after dialectical inquiry to reconcile brand perception of their employees [34].

This research study has focused on competitive advantage driven through employees and HR of firm/brand. This research study has identified the factors that cause employees to be involved in brand citizenship behavior and its impact on sustainable competitive advantage. The study demonstrated that the hotels must focus on the frontline non-managerial staff and ensure that the communication channels effectively communicate. Usually, low-level and non-managerial staff in organizations is not considered worthy of communication, but the results of this study reveal that these employees appreciate the contact and like to be well informed about the standards and values.

Another important factor of internal branding highlighted by this study is transformational leadership. This is the most appreciated factor by non-managerial staff [96]. A supervisor or manager, who is ’clear about his actions, encourages his/her subordinates, and gives them recognition would be considered an impressive person by non-managerial employees. His attitude and actions develop a sense of psychological commitment in his associates, leading them to show brand citizenship behavior inside and outside their organization. Through this behavior of employees, a hotel can deliver unique, different and better quality services than the market because when guests stay in the hotel, employees such as room attendants, phone operators, hosts and hostesses, receptionists, front desk officers, sales officers, bell captains, restaurants captains, barman, stewards, cashier and reservation agents deliver services to them, and create the perception of hotel’s values and standards in the mind of guests/customers. Therefore, managers need to understand the importance of non-managerial staff and their contribution in creating sustainable competitive advantage [13]. The competitors can imitate the financial and machinery assets, but imitating and implementing the same strategies and achieving similar employees’ behavior is very difficult [97]. Thus, a positive attitude and behavior can be observed in employees by implementing the internal branding strategy. Since a competitive advantage gained through this is not easy to imitate by the competitors, it makes it a sustainable competitive advantage for hotels.

## 7. Conclusion

In this era of intense competition, organizations need to look up new strategies to pursue sustainable competitive advantage. In the hotel industry, customer’s perception is based on the services they experience. Before making their positive perception about their experience at a hotel, it is essential to create a positive perception of those who deliver the services and create the perception of guests/customers. Since human resources play a vital role in providing services, the service industry should think differently about competitive advantage. Branding toward employees is as much crucial as branding focused on customers. According to the statistical results obtained in this research, it is clear that there is a significant and positive relationship between internal branding and sustainable competitive advantage. Brand commitment and brand citizenship behavior act as the critical mediators between internal branding and sustainable competitive advantage. The literature and the empirical evidence show that internal branding leads toward achieving sustainable competitive advantage through brand commitment and brand citizenship behavior.

## 8. Limitations and future recommendations

There are a few limitations of this study. The first limitation is that it is quantitative research in nature. However, it would be interesting for future researchers to qualitatively investigate the perception of frontline employees about internal branding because more and different internal branding factors can be observed with this approach. The second limitation is that the purposive sampling method used in this research has a generalizability issue. Future researchers may adopt different sampling techniques to collect data. The third limitation is that the present analysis is not comparative. However, future researchers may conduct a comparative study to compare the perception of managers and their subordinates about internal branding and brand citizenship behavior, and the comparison can also be performed among 3-, 4- and 5-star hotels. The fourth limitation is that only a few constructs have been taken as antecedents and the consequences of internal branding. Future studies should investigate other possible outcomes and relate them to customer-related values such as customer loyalty and customer satisfaction.

## Supporting information

S1 Dataset (RAR)
